# Publisher Correction: Inhibition of fatty acid oxidation enables heart regeneration in adult mice

**DOI:** 10.1038/s41586-023-06755-5

**Published:** 2023-10-20

**Authors:** Xiang Li, Fan Wu, Stefan Günther, Mario Looso, Carsten Kuenne, Ting Zhang, Marion Wiesnet, Stephan Klatt, Sven Zukunft, Ingrid Fleming, Gernot Poschet, Astrid Wietelmann, Ann Atzberger, Michael Potente, Xuejun Yuan, Thomas Braun

**Affiliations:** 1 Department of Cardiac Development and Remodeling, Max Planck Institute for Heart and Lung Research, Bad Nauheim, Germany; 2 Institute for Vascular Signaling, Centre for Molecular Medicine, Goethe-University, Frankfurt am Main, Germany; 3 Metabolomics Core Technology Platform, Centre for Organismal Studies (COS), Heidelberg University, Heidelberg, Germany; 4 Angiogenesis and Metabolism Laboratory, Max Planck Institute for Heart and Lung Research, Bad Nauheim, Germany; 5 Max Delbrück Center for Molecular Medicine, Helmholtz Association of German Research Centres, Berlin, Germany; 6 Instituto de Investigacion en Biomedicina de Buenos Aires (IBioBA) - CONICET - Partner Institute of the Max Planck Society, Buenos Aires, Argentina; 7 Present Address: Berlin Institute of Health, Charité-Universitätsmedizin Berlin, Berlin, Germany

**Keywords:** Histone post-translational modifications, Cardiac regeneration

Correction to: *Nature* 10.1038/s41586-023-06585-5 Published online 27 September 2023

In the version of the article initially published, there was an error in Fig. 2i where the arrows indicating tamoxifen treatment were shifted to the right. This has been corrected, and the arrows now start at day 1. There was also an error in the Fig. 5i *x*-axis labels, which mistakenly duplicated the labels of Fig. 5h. This has been corrected, and the *x*-axis labels now read “DMSO, αKG, *Kdm5b KD*, αKG + *Kdm5b KD*”. These changes have been made to the HTML and PDF versions of the article.

