# Supplementary material for: Inhibition of fatty acid oxidation enables heart regeneration in adult mice
Source: Nature. 2023 Sep 27;622(7983):619–26. doi: 10.1038/s41586-023-06585-5 (PMC10584682; doi:10.1038/s41586-023-06585-5)
Supplement: Supplementary file 1 — Supplementary Methods, References, Tables 1 and 2 and Figs. 1–7. [file 41586_2023_6585_MOESM1_ESM.pdf]

nature portfolio

https://doi.org/10.1038/s41586-023-06585-5

Supplementary information

Inhibition of fatty acid oxidation enables heart regeneration in adult mice

In the format provided by the authors and unedited

SUPPLEMENTARY INFORMATION

Inhibition of fatty acid oxidation enables heart regeneration in adult mice

Xiang Li[1], Fan Wu[1], Stefan Günther[1], Mario Looso[1], Carsten Kuenne[1], Ting Zhang[1], Marion Wiesnet[1], Stephan Klatt[2], Sven Zukunft[2], Ingrid Fleming[2], Gernot Poschet[3], Astrid Wietelmann[1], Ann Atzberger[1], Michael Potente[4,5], Xuejun Yuan[1,5*] and Thomas Braun[1,5,6*]

Table of contents:

Page 2-10: Supplementary Methods

Page 11: References for Supplementary Methods

Page 12-14: Supplementary Table1: List of primers used in this study

Page 15-16: Supplementary Table2: List of antibodies used in this study

Page 17-21: Supplementary Figure 1-5: Raw data for blots and gels

Page 22: Supplementary Figure 6: Full panels trichrome staining of heart sections after I/R

Page 23: Supplementary Figure 7: Gating strategy to sort PCM1 positive adult cardiomyocyte nuclei

METHODS

Animals.

Cpt1b$^{fl/fl}$ mice were generated in-house by using a targeting vector purchased from the European Conditional Mouse Mutagenesis Program (EUCOMM), in which exons 10-11 of the *Cpt1b* gene are flanked by two *LoxP* sites. Generation of *Ogdh*$^{fl/fl}$ animals, in which exons 3 and 4 were flanked by *loxP* sites, have been described before[26]. *αMHC-Cre*$^{Pos/+}$ and *αMHC-MCM*$^{pos/+}$ mice were obtained from The Jackson Laboratory. C57BL/6 mice were obtained from Charles River. Primers used for genotyping are listed in Supplementary Table 1. All mice were maintained in individually ventilated cages, at 22.5 °C ± 1 °C and a relative humidity of 50% ± 5% with controlled illumination (12 h dark/light cycle). Mice were given ad libitum access to food and water. Tamoxifen (Sigma) was administered intraperitoneally at 75mg/kg body weight daily for 10 days. In experiments, in which genes were inactivated by Cre recombinase-mediated recombination, corresponding Cre recombinase-expressing strains without the floxed target genes were always used as negative controls, unless indicated otherwise. *αMHC-MCM* control mice were subjected to the same tamoxifen treatment as in the actual gene inactivation experiment. All animal experiments were done in accordance with the Guide for the Care and Use of Laboratory Animals published by the US National Institute of Health (NIH Publication No. 85-23, revised 1996) and according to the regulations issued by the Committee for Animal Rights Protection of the State of Hessen (Regierungspraesidium Darmstadt, Wilhelminenstr. 1-3, 64283 Darmstadt, Germany) with the project number B2/1137, B2/1125, B2/2034.

Neonatal CM isolation and culture *in vitro*

Neonatal hearts were dissected from P0-1 C57bl/6 pups, washed with ice-cold PBS, and dissociated using standard procedures. Neonatal CMs were seeded in fibronectin pre-coated culture plates (0.8-1 million per 3.5 dish or 0.25 to 0.4 million per well of 2-well chamber slides) and cultured in primary neonatal CM culture medium (80% DMEM with 4.5 g/l glucose, 20% Medium 199, 5% FCS, 100 U/ml penicillin/streptomycin). After overnight culture, chemicals were added to the medium and cells were further cultured for 72 to 96 hours before harvest. Concentrations of chemical are as follows: etomoxir 100μM (Cayman, 11969); octyl-α-ketoglutarate 500μM (Cayman, 11970); CPI-HCl 25μM (Selleckchem, S8287); R2HG 500μM (Cayman, 16366). *Kdm5b* knockdown was achieved via transfection of pooled *Kdm5b* siRNA (Dharmacon) with DharmaFECT 1 Transfection Reagent. Non-targeting pooled siRNAs were used for the Ctrl group.

Immunofluorescence and histological analysis.

Hearts were immediately fixed in 4% PFA after dissection. For trichrome staining after I/R injury, hearts were embedded in paraffin and continuously sectioned from the apex to the ligation site. Every second section from hearts of each group was used for staining and quantification. *In vitro* cultured neonatal CMs were fixed with 4% PFA for 10min at room temperature and permeabilized (0.3% Triton X-100 and 5% BSA) for 1 hour at room temperature. To determine surface area of CMs, approximately randomly selected 120 CMs from 5-6 paraffin sections of each heart sample were measured using the Image J software tool. The sarcomere density of isolated adult CM was analyzed using the Image J software tool. For other quantifications, either using isolated CMs or tissue sections, hundreds of CMs were analyzed for each individual heart. Values from each group of CMs (or sections) were averaged and are presented as one sample (n=1). For experiments with neonatal CMs, values for each sample represent the results obtained from one isolation of CMs from pooled neonatal hearts. Antibodies for immunofluorescence staining are listed in Supplementary Table 2. Microscopic images were acquired with a fluorescence stereomicroscope (Leica M205 FA). Immunoflurescence images were acquired with a fluorescence microscope (ZEISS Imager Z1)

and Leica SP8 Confocal microscope. Acquisition of histological image was performed with a light microscope (Zeiss Axioplan2). Image processing was done with ZEN 2 Imaging Software, Leica SP8 - software LAS X 3.5.7.23225.

EdU incorporation assay

EdU and other reagents were prepared according to the manufacturer's instruction (ThermoFisher C10339). *In vivo* EdU incorporation assays were done according to previous publications[45]. To analyze EdU incorporation in cultured neonatal CMs, cells in 2-well chamber slides were labeled with 10 µM EdU for 12 hrs. After two times washing with pre-warmed PBS, cells were fixed with 4% PFA for 10min at room temperature and EdU incorporation was visualized using the Click-iT EdU kit (Invitrogen), following the manufacturer's protocol.

Western blot assays

Freshly isolated or cultured cells were washed with ice-cold PBS and lysed in cell lysis buffer (20 mM Tris (pH 7.5), 400 mM NaCl, 1 mM EDTA, 1 mM EGTA, 1% Triton X-100, 1× Complete Protease Inhibitor Cocktail (Roche Diagnostics)) for 10 min on ice, followed by sonication using the Bioruptor (Dianagene) at 4°C for 5 min. Proteins were separated by SDS-PAGE and transferred to nitrocellulose membranes (Millipore, Billerica, MA). Proteins detected by antibodies were visualized using an enhanced chemiluminescence detection system (GE Healthcare) and quantified using the ChemiDoc gel documentation system (Bio-Rad). Antibodies and dilutions used in this study are listed in Supplementary Table 2.

Adult cardiomyocyte isolation and *in vitro* culture

Isolation of adult CMs was performed as described previously[46]. In brief, dissected hearts were cannulated via the aorta and retrogradely perfused with calcium-free buffer. Cannulated hearts were enzymatically digested by perfusion with enzyme buffer solution and cut off from the cannula. Atria were separated, and ventricles were minced in Enzyme Buffer. After gentle pipetting, myocytes were centrifuged at 500 rpm for 1 min and cell pellets containing CM fractions were re-suspended in Stop Buffer. The calcium content of the cell suspension was then stepwise adjusted to 1 mM and CM-containing cell pellets were re-suspended in M199 cell culture medium, supplemented with creatinine, L-carnitine, HEPES, penicillin/streptavidin, 5% FCS, and insulin-transferrin-sodium selenite media supplement. Cells were seeded in laminin pre-coated dishes and maintained in a humidified incubator at 37°C and 5 % CO_2. To determine CM numbers in adult hearts, the dissected heart was washed with ice-cold PBS and fixed with 1% PFA overnight. After washing with ice-cold PBS, hearts were cut into 1-2mm^3 pieces and incubated with digestion buffer (PBS containing 0.5U/ml collagenase B (Roche #11088807001) and 0.2% NaN_3) with constant shaking at 1000 rpm at 37°C. Every 12-24 hours, digested CM was collected, and new digestion buffer was added until the heart was fully digested. CMs were pooled, plated into a Sedgewick rafter chamber, and counted therein.

Oxygen consumption rate (OCR) measurement with Agilent Seahorse XF

Adult CMs were isolated and seeded at a density of 6000 cells per well in a 96 well plate for Seahorse measurements (Agilent Seahorse XFe96 Analyzer). Cells were washed with PBS and Seahorse base medium after attachment to the plate. The following substrates were added as energy substrates to the medium 1 hour before OCR measurements: Glucose 5mM (Sigma), Pyruvate 0.2mM (Sigma), Glutamine 4mM (Sigma), Palmitate-BSA 0.2mM (Agilent), BSA Ctrl (Agilent), Carnitine 0.2mM (Sigma), Valine 1mM (Sigma), Isoleucine 1mM (Sigma), Leucine 1mM (Sigma), Sodium propionate 0.05mM (Sigma), Sodium acetate 0.05mM (Sigma), Sodium octanoate 0.1mM (Sigma), Sodium decanoate 0.1mM (Sigma). The OCR was measured using the Mito Stress Test kit (Agilent). The following inhibitors were injected: Oligomycin (2µM), FCCP (2µM), Rotenone and antimycin A (1µM).

MRI and data processing
Cardiac MRI measurements were performed using a 7.0 T Bruker Pharmascan (Bruker, Ettlingen, Germany) equipped with a 760 mT/m gradient system, using a cryogenically cooled 4 channel phased array element 1H receiver-coil (CryoProbe), a 72 mm room temperature volume resonator for transmission, and the IntraGateTM self-gating tool[47]. ECG parameters were adapted for one heart slice and transferred afterwards to the navigator signals of the remaining slices. Thus, in-phase reconstruction of all pictures was guaranteed. Measurements are based on the gradient echo method (repetition time = 6.2 ms; echo time = 1.3 ms; field of view = 2.20x2.20 cm; slice thickness = 1.0 mm; matrix = 128 x 128; oversampling = 100). The imaging plane was localized using scout images showing the 2- and 4-chamber view of the heart, followed by acquisition of images in short axis view, orthogonal on the septum in both scouts. Multiple contiguous short-axis slices consisting of 7 to 10 slices were acquired for complete coverage of the left and right ventricle. Mice were measured under isoflurane (1.5 – 2.0 % in oxygen and air with a flow rate of 1.0 l/min) anesthesia. Body temperature was maintained at 37°C by a thermostatically regulated water flow system during the entire imaging protocol. MRI data were analyzed using Qmass digital imaging software (Medis Imaging Systems, Leiden, Netherlands).

FACS-based isolation of cardiac nuclei
Ventricles were washed with ice-cold PBS after dissection and snap frozen in liquid N_2. For cardiac nuclei isolation, the frozen ventricle was thawed in 3ml lysis buffer (5 mM $CaCl_2$, 3 mM MgAc, 2 mM EDTA, 0.5 mM EGTA, and 10 mM Tris-HCl, pH 8) in M-tubes (Miltenyi Biotec) and homogenized using the gentleMACS Dissociator (Miltenyi Biotec), following the manufacturer's protocol (protein_01). The resultant homogenate was mixed with lysis buffer containing 0.4% Triton X-100, incubated on ice for 10 min, and subsequently filtered through 40µm cell strainers (BD Bioscience). The flow-through was subjected to centrifugation at 1000 g for 5min at 4°C to harvest nuclei. Nuclei were further purified by centrifugation at 1000g for 5 min at 4°C through a 1M sucrose cushion (3 mM MgAc, 10 mM Tris-HCl, pH8) and then stained with a PCM1 antibody in nuclei stain buffer (DPBS, 1% BSA, 0.2% Igepal CA-630, 1 mM EDTA). DNA was stained by DAPI before FACS. FACS was done using a FACSAria^{TM} III (BD Biosciences). Quantification of PCM^{+} cardiac nuclei and DNA content was performed with the LSR Fortessa (BD Biosciences) analyzer. Data acquisition and analysis were accomplished using the BD FACS Diva v8 software. The gating strategy is shown in Supplementary Figure 7.

RNA-seq and data analysis
RNA was extracted from isolated adult CMs using the Direct-zol Total Kit (Zymo Research) combined with on-column DNase digestion (DNase-Free DNase Set, Qiagen) to avoid contamination by genomic DNA. RNA and library preparation integrity were verified using the LabChip Gx Touch 24 (Perkin Elmer). 200ng of total RNA was used as input for the SMARTer Stranded Total RNA Sample Prep Kit - HI Mammalian (Clontech) following the manufacturer's instructions. Sequencing was performed on a NextSeq500 instrument (Illumina) using v2 chemistry, resulting in an average of 22M reads per library with 1x75bp single end setup. Raw reads were assessed for quality, adapter content and duplication rates with FastQC 0.11.8 (http://www.bioinformatics.babraham.ac.uk/projects/fastqc). Trimmomatic version >=0.36 was employed to trim reads after a quality drop below a mean of Q15 in a window of 5 nucleotides[48]. Only reads of at least 15 nucleotides were cleared for subsequent analyses. Trimmed and filtered reads were aligned versus mouse genome version mm10 (GRCm38.p5) using STAR >=2.5.4b with the parameters "--outFilterMismatchNoverLmax 0.1 --alignIntronMax 200000" [49]. The number of reads aligning to genes was counted with

featureCounts >=1.6.0 from the Subread package[50]. Only reads mapping at least partially inside exons were admitted and aggregated per gene. Reads overlapping multiple genes or aligning to multiple regions were excluded. Differentially expressed genes were identified using DESeq2 version >=1.14.0[51]. Genes were classified to be significantly differentially expressed (DEG) with a P-Value < 0.05. Annotations were enriched using UniProt data (release 24.03.2017) based on Ensembl gene identifiers (Activities at the Universal Protein Resource (UniProt)).

Chromatin immunoprecipitation (ChIP), ChIP-seq, and data analysis
Chromatin was prepared using the truChIP Chromatin Shearing Kit (COVARIS) and sheared to an average size of 200–500 bp by sonication (Diagenode Bioruptor). Protein-DNA complexes were immunoprecipitated with IgG or KDM5B antibodies, followed by incubation with Protein A/G magnetic beads (Dynabeads, Invitrogen). For ChIP-qPCR, beads were washed and protein-DNA complexes were eluted and purified using 10% chelex-100 (w:v, Bio-Rad Laboratories) in Tris-EDTA. Immunoprecipitated chromatin was analyzed by qPCR using SYBR Green quantitative real time analysis with primers that are listed in Supplementary Table 1. ChIP-seq experiments were performed according to standard protocols. Briefly, FACS-purified cardiac nuclei were first cross-linked with 1% formaldehyde for 5 min and then quenched using reagents from the truChIP Chromatin Shearing Kit (COVARIS) for 10 min at RT. Chromatin was sheared to an average size of 200–500 bp by sonication (Diagnode Biorupter). Protein-DNA complexes were eluted from beads by incubation with 50 µl elution buffer (10 mM Tris-HCl pH 7.4, 5 mM EDTA, 300 mM NaCl, 0.5% SDS) at RT for 5 min and treated with 1 µg DNase free-RNase (Roche) at 37ºC for 30 min. After incubation with 25 µg of proteinase K (10 mg/ml), 1 µg Glycogen at 37ºC for 2 hours, samples were heated at 65ºC with constant shaking at 1350 rpm overnight. DNA was purified using the MinElute® PCR purification Kit (Qiagen) and quantified by the Qubit dsDNA HS Assay Kit (Thermo Fisher Scientific). 0.5-10 ng of DNA was used as input for the TruSeq ChIP Library Preparation Kit (Illumina) with the following modifications: libraries were size selected by the SPRI-bead based approach after final PCR with 18 cycles instead of gel-based size selection before the final PCR step. Samples were first cleaned-up with a 1× bead:DNA ratio to eliminate residuals from the PCR reaction, followed by a 2-sided-bead cleanup step with a 0.6× bead:DNA ratio to exclude larger fragments. Supernatants were transferred to new tubes and incubated with additional beads using a 0.2× bead:DNA ratio to eliminate small fragments, such as adapters and primer dimers. Bead-bound DNA was washed with 80% ethanol, dried and re-suspended in TE buffer. Library integrity was verified with the LabChip Gx Touch 24 instrument (Perkin Elmer). Sequencing was performed on a NextSeq500 instrument (Illumina) using v2 chemistry with a 1×75bp single end setup.
Raw reads were assessed for quality, adapter content and duplication rates with FastQC 0.11.8 (http://www.bioinformatics.babraham.ac.uk/projects/fastqc). Trimmomatic version 0.39 was employed to trim reads at a quality drop below a mean of Q15 in a window of 5. Only reads of at least 15 nucleotides were cleared for subsequent analyses. Trimmed and filtered reads were aligned versus mouse genome version mm10 (GRCm38) using STAR 2.6.1d with the parameters "--outFilterMismatchNoverLmax 0.1 --outMultimapperOrder Random --outSAMmultNmax 1 --outFilterMultimapNmax 1 --alignEndsType EndToEnd --alignIntronMax 1". These options exclude spliced and multi-mapping alignments. Reads were further de-duplicated using Picard 2.21.7 (https://github.com/broadinstitute/picard/releases/tag/2.21.7) to reduce PCR artefacts leading to multiple copies of the same original fragment. The MUSIC peakcaller (version of Dec. 2015) was employed in punctate mode to identify enriched regions when comparing the respective ChIP to input samples. MUSIC FDR was set to 0.2. Peaks overlapping ENCODE blacklisted regions (known misassemblies, satellite repeats) were excluded. In order to compare peaks in different samples for assessment of reproducibility, the resulting lists of significant peaks were

overlapped and unified to represent identical regions. Sample counts for union peaks were produced using bigWigAverageOverBed (UCSC Toolkit) and normalized with DESeq2 1.26.0 to compensate for differences in sequencing depth, library composition, and efficiency. Peaks were annotated with the promoter of the nearest gene (TSS +- 5000 nt) using reference data of GENCODE vM15. Peaks were classified to be significantly differentially present with P-Value < 0.05 as produced by DESeq2. Peaks visualization was done with IGV 2.3.52. Peaks were divided into 3 groups based on peak widths (Broad: >75th %, Medium: 75th – 25th %, Narrow: <25th %). DESeq normalized peak counts were log2-transformed and used as input for a One-way ANOVA analysis with Graphpad Prism 9. Pairwise comparisons were corrected for multiple testing by controlling the FDR with the two-stage step-up method of Benjamini, Krieger and Yekutieli.

Ischemia/Reperfusion (I/R) injury and measurement of area at risk (AAR) and infarct area out of AAR

Animals were anesthetized using 4.5% isoflurane and subjected to endotracheal intubation with a 22 Gauge intravenous catheter. Mice were placed on a 37°C heating plate in the supine position and ventilated at a rate of 225 strokes/min and a stroke volume of 250 µl with a mixture of oxygen and 1.5% isoflurane using a MiniVent rodent ventilator. Chest hair was removed, skin disinfected and opened with a small incision of several mm length from the left armpit to the sternal border. Pectoralis major and minor muscles were separated, the chest was opened in the 3rd intercostal space, and retractors were inserted. Next, the pericardium was opened to access the heart. The left coronary artery was ligated for 30min and reopened for reperfusion in a proximal position using a prolene suture (7-0). The retractors were removed, the chest wall was closed by bringing together the 2rd and 3th rib using a vicryl suture (5-0). The muscles were placed into their original position and the skin incision was closed with vicryl (5-0). Mice were ventilated with oxygen until awakening, followed by extubation, and placement into their cages. 24 hours after I/R surgery, the animals were scarified for AAR and infarct area out of AAR measurement, for which hearts were removed and the aorta quickly cannulated for an injection of 500µl 1% Evan's Blue solution into the ventricle. Hearts were kept on ice cold saline for further investigations. Afterward, the heart was frozen and sliced at 0.5mm. Heart sections were subsequently stained with TTC solution (1% in PBS) at 37 °C for 30min and then fixed with formalin solution.

Viability assay of adult CM under hypoxic condition

Freshly isolated adult CMs seeded in chamber slides were cultured either under normoxia or in a hypoxia chamber with 1% O_2, 5% CO_2 at 37 °C for 18 hours. Cells were washed at room temperature with PBS and incubated with PBS containing EthD1 (4 µM) and calcineurin (2 µM) for 45 min at RT. Immunofluorescence images were acquired with a Zeiss Imager Z1 microscope.

Metabolic flux assays and targeted metabolic analysis

Metabolic flux assays were performed with isolated Langendorff-perfused hearts from *Ctrl*^{Cre} and *Cpt1b*^{cKO} mice. Hearts were quickly excised and cannulated via the aorta. The cannulated heart was connected to a perfusion column apparatus maintained at 37 °C using a temperature-controlled water bath. Hearts were perfused retrogradely for 60 min with Krebs-Henseleit (KH) buffer with the following substrates: Glucose 8 mM; Pyruvate 0.12 mM; Palmitate-BSA 0.4 mM; Isoleucine 0.176 mM. In each perfusion assay, only one metabolite was replaced by a ^{13}C-labelled metabolite (^{13}C-Glucose, ^{13}C-Isoleucine or ^{13}C-Palmitate-BSA). Subsequently, hearts were snap-frozen, pulverized in liquid nitrogen and subjected to metabolite extraction (acyl-CoAs or metabolites of the Krebs cycle) and quantification via LC-MS QQQ. Metabolic flux assays and α-ketoacids measurements were done using tissue from isolated hearts after

perfusion with different substrates as indicated. Measurements of Krebs Cycle metabolites and standardized targeted metabolic analysis were done with isolated adult CMs.

Quantification/Analysis of acyl-CoAs from ^{13}C-perfused heart

Pulverized heart samples were solubilized in 20 µl/mg of 10% trichloroacetic acid in water. Samples were shortly vortexed and sonicated in an ice-bath for 30 sec. Sonication was repeated 5-6-times until homogenization was reached. To remove proteins and insoluble material, samples were centrifuged at 14000 rpm at 4°C for 10min. Next, samples were subjected to solid phase extraction (SPE) via the *Waters Positive Pressure-96* Processor, required to remove salts and phospholipids. Supernatants were directly loaded onto Oasis PRiME HLB plates (Waters; 1cc, 30 mg sorbent per well). The plates were washed with 1 mL of MilliQ water and bound acyl-CoAs were eluted with 650 µL of 100% methanol (containing 25 mM ammonium acetate). The eluate was diluted with 2-times the volume of MilliQ water, frozen at -80 °C and subsequently freeze-dried using the Alpha 3-4 LSCbasic system (Martin Christ, Osterode am Harz/Germany). Dried samples were reconstituted in 80 µL of 85% acetonitrile (AcN:H_2O, 85:15, v/v), centrifuged at 14000 rpm at 4 °C for 10 min, transferred to MS glass vials, and subjected to mass spectrometric analysis.

In detail, 5 µl of each sample was injected via an Infinity II Bio liquid chromatography system into a 6495C triple quadrupole mass spectrometer (both Agilent Technologies, Waldbronn/Germany). Acyl-CoAs were separated on an InfinityLab Poroshell 120 HILIC-Z column (2.1x150 mm, 2.7 µm; Agilent Technologies) by using the following mobile phase binary solvent system and gradient at a flow-rate of 0.4 mL/min: Mobile A consisted of 100% water with 20 mM ammonium acetate and 5 µM medronic acid at a pH of 9.3. Mobile phase B consisted of 100% acetonitrile. The following 27 min gradient program was used: 0 min 85% B, 0-1 min 85% B, 1-8 min 75% B, 8-12 min 60% B, 12-15 min 10% B, 15-18 min 10% B, 18-19 min 85% B, 19-27 min 85% B. The column compartment was set to 15 °C. Metabolites were detected with authentic standards and/or via their accurate mass, fragmentation pattern and retention time in positive ionization dynamic MRM AJS-ESI mode, and quantified (where appropriate) via a calibration curve. The isotopologues of each acyl-CoA were detected by using the acyl-specific fragment and the influx of 2-^{13}C units into the acyl backbone. For example, the following isotopologues of octanoyl-CoA were included: octanoyl-8-^{12}C-CoA, octanoyl-2-^{13}C-CoA, octanoyl-4-^{13}C-CoA, octanoyl-6-^{13}C-CoA and octanoyl-8-^{13}C-CoA. The gas temperature of the mass spectrometer was set to 200 ºC and the gas flow to 14 L/min. The nebulizer was set to 40 psi. The sheath gas flow was set to 12 L/min, with a temperature of 375ºC. The capillary voltage was set at 4000 V with a nozzle voltage of 500 V. The voltages of the High-Pressure RF and Low-Pressure RF were set to 150/60 V, respectively.

Metabolite peaks were annotated with Skyline-daily (version 22.2.1.278) and the concentration was calculated with GraphPad Prism 9.

Quantification/Analysis of Krebs cycle metabolites from the ^{13}C-perfused heart

Pulverized heart samples were solubilized in 20 µl/mg of methanol extraction buffer (containing 1 mM TCEP, 1mM ascorbic acid and 0.1% formic acid in 85:15 methanol:H_2O; with the internal standard of dimethyl-3-hydroxyglutarate at a concentration of 0.1 mM). Samples were shortly vortexed and sonicated in an ice-bath for 30 sec. Sonication was repeated 6-8-times until homogenization was reached, and the homogenate was further incubated on ice for 10 min. Next, samples were centrifuged at 14000 rpm at 4°C for 10min to remove protein and insoluble material. Supernatants were mixed with 2-times the volume of MilliQ water, frozen at – 80 °C, and subsequently freeze-dried (as above). Dried samples were reconstituted in 50 µL of 0.1% formic acid in MilliQ water, centrifuged at 14000 rpm at 4°C for 10 min, transferred to MS glass vials, and subjected to mass spectrometric analysis.

In detail, 5 µl of each sample was injected via an Infinity II Bio liquid chromatography system into a 6495C triple quadrupole mass spectrometer (both Agilent Technologies, Waldbronn/Germany). Metabolites were separated on an Acquity UPLC HSS T3 column (RRHD 2.1x150mm, 1.8µm; Waters) by using the following mobile phase binary solvent system and gradient at a flow-rate of 0.3 mL/min: Mobile A consisted of 100% water with 0.1% formic acid and mobile phase B consisted of 100% acetonitrile with 0.1% formic acid. The following 15 min gradient program was used: 0 min 1% B, 0-2 min 1% B, 2-8 min 50% B, 8-8.1 min 99% B, 8.1-11 min 99% B, 11-11.1 min 1% B, and 11.1-15 min 1% B. Further, there was a post-time of 2 min at 1 % B. The column compartment was set to 40°C. Metabolites were detected with authentic standards and/or via their accurate mass, fragmentation pattern and retention time in negative ionization dynamic MRM AJS-ESI mode, and quantified (where appropriate) via a calibration curve. The isotopologues of each metabolite were detected by including partially to fully ^{13}C-labelled fragments. The gas temperature of the mass spectrometer was set to 240 ºC and the gas flow to 19 L/min. The nebulizer was set to 50 psi. The sheath gas flow was set to 11 L/min, with a temperature of 400ºC. The capillary voltage was set at 1000 V with a nozzle voltage of 500 V. The voltages of the High-Pressure RF and Low-Pressure RF were set to 100/70 V, respectively. Metabolite peaks were annotated with Skyline-daily (version 22.2.1.278) and the concentration was calculated with GraphPad Prism 9.

Quantification of Krebs Cycle metabolites
Isolated CM were homogenized in 85% methanol (4 µl/10,000 cells), and the homogenate was centrifuged at 10,000 g for 5 minutes at 4°C. An equal volume of supernatant was collected and isotope labeled internal standard were added. The samples were dried by evaporation in a ConcentratorPlus (Eppendorf, Wesseling-Berzdorf, Germany) and were reconstituted in 50 µl water, transferred to autosampler vials and subsequently analyzed by LC-MS/MS.
Liquid chromatography was performed on an Agilent 1290 Infinity LC system (Agilent, Waldbronn, Germany). Reversed-phase separation was performed using a Waters Acquity UPLC HSS T3 column (150 mm × 2.1 mm, 1.8 µm) at 40°C. Gradient elution was performed with 0.15% formic acid in water (mobile phase A) and 0.15% formic acid in acetonitrile (mobile phase B) at a flow rate of 0.4 mL/min. Gradient conditions were 2% B for 1.5 min, followed by a 3 min gradient to 100% B, followed by a cleaning and equilibration step, resulting in a 10 min total LC run time. Injection volume was 2.5 µl for all samples. Autosampler temperature was set to 6°C. Mass spectrometry was performed on a QTrap 5500 mass spectrometer (Sciex, Darmstadt, Germany) equipped with an ESI TurboIonSpray source. Electro spray ionization was set to 400°C and -4500 V in negative ionization mode was employed. Ion source gas parameters were as follows: CUR 30 psi, GS1 45 psi, GS2 25 psi. The specific MRM transition for every compound was normalized to appropriate isotope labeled internal standards. Calibration curves were performed with authentic standards. Analyst 1.6.2 and MultiQuant 3.0 (both from Sciex, Darmstadt, Germany) were used for data acquisition and analysis, respectively.

Standardized metabolic profiling using the AbsoluteIDQ® p400 HR and the MxP® Quant p500 kits
Two standardized metabolic profiling kits from Biocrates to quantify more than 400 (p400) and up to 630 (p500) metabolites respectively. In brief, isolated CM were homogenized in 85% methanol (4 µl/10,000 cells), and the homogenate was centrifuged at 10000 g for 5 minutes at 4°C. Next, 10 µL of the supernatants (samples and blanks) were transferred onto the provided 96-well plates and processed according to the manufacturer's protocols (p400 and p500 kits). Each 96-well plate also contained calibrators and internal standards. Samples were dried under nitrogen in a TurpoVap (Biotage, Uppsala, Sweden) for 30 minutes. Next, 50 µL of a 5% phenyl

isothiocyanate solution was added to each well to derivatize amino acids and secondary amines. The plates were incubated at RT for 20 min (p400) or 60 min (p500), and then dried again under nitrogen stream. Next, 300 µL of a 5 mM ammonium acetate in methanol solution was added to each well. The 96-well plates were incubated at RT for 30 min on a shaker at 450 rpm, followed by a 2 min centrifugation step at 500 g. For LC-MS/MS analysis, 150 µL of the eluate was transferred to a new 96-well plate and diluted 1:1 with MilliQ water. For the FIA-MS/MS analysis, 250 µL (p400) and 490 µL (p500) of FIA solvent (Biocrates) were added to 10 µL of each extract. All plates were shaken for 5 min at 450 rpm.
LC-MS/MS and FIA-MS/MS measurements were performed in the following way: P400: Metabolites were analyzed by using an Accela UHPLC system coupled to a Q-Exactive classic (both Thermo Fisher Scientific, Dreieich/Germany). 5 µl of each sample was injected onto a Biocrates Absolute IDQ column. The mobile phases for the LC-MS/MS set-up consisted of 0.2% formic acid in MilliQ water (solvent A) and 0.2% formic acid in acetonitrile (solvent B). The following gradient was used at a flow rate of 860 µl/min: 0-0.25 min 0% B, 1.5 min 12% B; 2.7 min 17.5% B; 4 min 50% B; 4.50 min 95% B; 5.25-5.80 min 0% B. The column oven temperature was set to 50°C. Xcalibur 4.0 software (Thermo Scientific, Dreieich, Germany) was used for data acquisition and calculation of molar concentrations. For FIA-MS/MS analysis, 20 µl per sample was directly injected into the MS, with an isocratic flow of methanol containing 3.33% Biocrates Solvent I at a flow rate of 95 µl/min. Data evaluation for LC- and FIA-methods was performed with the MetIDQ 6.0 software (Biocrates, Innsbruck/Austria). P500: Here, metabolites were analyzed by using a 1290 Infinity LC system (Agilent Technologies, Waldbronn, Germany) coupled to a QTrap 5500 mass spectrometer (Sciex, Darmstadt/Germany). LC-/FIA gradient and mass spectrometric parameters were set according to the manufacturer's protocols, and metabolites were analyzed using internal standards and the MRM settings provided by Biocrates.

Quantification of branched-chain α-ketoacids
To measure branched-chain α-ketoacids, 30 mg of homogenized heart tissue was extracted with 300 µl cold 1M perchloric acid. Insoluble material was removed by centrifugation for 10 min at 25.000g. For derivatization with DMB (1,2-diamino-4,5-methylendioxybenzene), 30 µl extract were mixed with 30 µl DMB reagent (5mM DMB, 20mM sodium hydrosulfite, 1M 2-mercaptoethanol, 1.2M HCl) and incubated at 100°C for 45 min. After 10 min centrifugation, the reaction was diluted with 240 µl 10% acetonitrile and the derivatized ketoacids were separated by reversed phase chromatography on an Acquity HSS T3 column (100 mm x 2.1 mm, 1.7 µm, Waters) connected to an Acquity H-class UPLC system. The column was heated to 40°C prior to separation and equilibrated with 5 column volumes of solvent A (0.1% formic acid and 10% acetonitrile) at a flow rate of 0.55 ml/min. Baseline separation of DMB derivates was achieved by increasing the concentration of acetonitrile (B) in buffer A as follows: 2 min 2% B, 4.5 min 15% B, 10.5 min 38% B, 10.6 min 90% B, hold for 2 min, and return to 2% B in 3.5 min. The separated derivates were detected by fluorescence (Acquity FLR detector, Waters, excitation: 367 nm, emission: 446 nm) and quantified using ultrapure standards (Sigma). Data acquisition and processing was performed with the Empower3 software suite (Waters).

Lentiviral transduction of CMs
HEK293T cells were grown in DMEM (Sigma) supplemented with 10% FCS (Sigma), 2mM L-Glutamine, 100U Penicillin, and 100 µg /ml Streptomycin at 37°C, 5% CO_2. HEK293T cells ($2x10^6$/10 cm dish) were transfected with 5 µg pLJM1-Kdm5b, or pLJM1-Idh3b or pLJM1-Idh3g, 4.5 µg psPAX2 (Addgene, #12260), and 0.5 µg pMD2.G (Addgene, #12259) using the Turbofect transfection reagent and Opti-MEM™ for 6-8 hours. The supernatants containing lentiviral particles were collected at 48 and 72 hours after transfection and pooled. Lentiviruses

were filtered through a 0.45 µM cell strainer to remove HEK293T cells and concentrated with a Lenti-X concentrator according to the manufacturer's instruction (TaKaRa, 631231). Primary neonatal CMs were infected in suspension with Polybrene (8µg/ml) for 6 to 8 hours.

RT-qPCR gene expression analysis and assessment of mitochondrial DNA copy numbers
Total RNA was extracted using the TRIzol reagent (Invitrogen) according to the manufacturer's instruction. RNA was reverse-transcribed with Superscript II (Invitrogen) following standard procedures. Real-time PCR was performed with 2 technical replicates using StepOne^{TM} Real time PCR system and KAPA SYBR ® FAST qPCR Master Mix (KAPA Biosystems, CH). Relative quantitation of gene expression was performed using the ΔΔCT method. The Ct values of the target genes were normalized to expression of the *36b4* gene using the equation $\Delta Ct = Ct_{reference} - Ct_{target}$ and expressed as ΔCt. Relative mRNA expressions were shown with the average from control samples set as 1. Mitochondrial DNA copy numbers were determined using DNA extracted from isolated adult CMs. The data were normalized to internal controls (*H19* or *Mx1*) and cell numbers. Primers and PCR conditions are listed in Supplementary Table 1.

Electron microscopy
Hearts were isolated and fixed in 1.5% glutaraldehyde (v/v), 1.5% paraformaldehyde (v/w) in 0.15 M HEPES (v/w), pH 8.0 at 4 °C for at least 24 h, and subsequently incubated with 1 % osmium tetroxide for 2 hours. Samples were stained en bloc with 50 % saturated watery uranyl acetate, followed by sequential ethanol dehydration (30%, 50%, 75%, 95%), and embedded in Agar 100. Ultrathin sections were cut using an ultramicrotome and image acquisition was performed with a Philips CM10 electron microscope. All images were captured with a slow-scan 2K CCD camera.

Statistical analysis.
For all quantitative analyses, a minimum of three biological replicates was analyzed. Statistical tests were selected based on the assumption that sample data are from a population following a probability distribution based on a fixed set of parameters. Student's t-tests were used to determine the statistical significance of differences between two groups. One-way AVOVA was used for multiple comparison tests. The following values were considered to be statistically significant: *P<0.05, **P<0.01, P***<0.001, P****<0.0001. Calculations were done using the GraphPad Prism 9 software package. Data are always represented as mean ± the standard error of the mean. No statistical method was used to predetermine sample size.

References for Methods:

45 Richardson, G. D. Simultaneous Assessment of Cardiomyocyte DNA Synthesis and Ploidy: A Method to Assist Quantification of Cardiomyocyte Regeneration and Turnover. *J Vis Exp* (2016). https://doi.org/10.3791/53979

46 O'Connell, T. D., Rodrigo, M. C. & Simpson, P. C. Isolation and culture of adult mouse cardiac myocytes. *Methods Mol Biol* **357**, 271-296 (2007). https://doi.org/10.1385/1-59745-214-9:271

47 Larson, A. C. *et al.* Self-gated cardiac cine MRI. *Magn Reson Med* **51**, 93-102 (2004). https://doi.org/10.1002/mrm.10664

48 Bolger, A. M., Lohse, M. & Usadel, B. Trimmomatic: a flexible trimmer for Illumina sequence data. *Bioinformatics* **30**, 2114-2120 (2014). https://doi.org/10.1093/bioinformatics/btu170

49 Dobin, A. *et al.* STAR: ultrafast universal RNA-seq aligner. *Bioinformatics* **29**, 15-21 (2013). https://doi.org/10.1093/bioinformatics/bts635

50 Liao, Y., Smyth, G. K. & Shi, W. featureCounts: an efficient general purpose program for assigning sequence reads to genomic features. *Bioinformatics* **30**, 923-930 (2014). https://doi.org/10.1093/bioinformatics/btt656

51 Love, M. I., Huber, W. & Anders, S. Moderated estimation of fold change and dispersion for RNA-seq data with DESeq2. *Genome Biol* **15**, 550 (2014). https://doi.org/10.1186/s13059-014-0550-8

SUPPLEMENTARY TABLES

Supplementary Table 1: List of primers used in this study

Name	Primer sequence (5'-->3')	Application
Slc2a1 qF	CAGTTCGGCTATAACACTGGTG	qPCR
Slc2a1 qR	GCCCCCGACAGAGAAGATG	qPCR
Slc2a4 qF	GTGACTGGAACACTGGTCCTA	qPCR
Slc2a4 qR	CCAGCCACGTTGCATTGTAG	qPCR
Ldhb qF	CATTGCGTCCGTTGCAGATG	qPCR
Ldhb qR	GGAGGAACAAGCTCCCGTG	qPCR
Pfkm qF	TGTGGTCCGAGTTGGTATCTT	qPCR
Pfkm qF	GCACTTCCAATCACTGTGCC	qPCR
Cpt1a qF	CTCCGCCTGAGCCATGAAG	qPCR
Cpt1a qR	CACCAGTGATGATGCCATTCT	qPCR
Cpt1b qF	TCTAGGCAATGCCGTTCAC	qPCR
Cpt1b qR	GAGCACATGGGCACCATAC	qPCR
Acta1 qF	CCCAAAGCTAACCGGGAGAAG	qPCR
Acta1 qR	CCAGAATCCAACACGATGCC	qPCR
Nppa qF	GCTTCCAGGCCATATTGGAG	qPCR
Nppa qR	GGGGGCATGACCTCATCTT	qPCR
Nppb qF	GAGGTCACTCCTATCCTCTGG	qPCR
Nppb qR	GCCATTTCCTCCGACTTTTCTC	qPCR
Ldha qF	TGTCTCCAGCAAAGACTACTGT	qPCR
Ldha qR	GACTGTACTTGACAATGTTGGGA	qPCR
Atp2a2 qF	GAGAACGCTCACACAAAGACC	qPCR
Atp2a2 qR	CAATTCGTTGGAGCCCCAT	qPCR
Pln qF	AAAGTGCAATACCTCACTCGC	qPCR
Pln qR	GGCATTTCAATAGTGGAGGCTC	qPCR
Slc8a1 qF	CTTCCCTGTTTGTGCTCCTGT	qPCR
Slc8a1 qR	AGAAGCCCTTTATGTGGCAGTA	qPCR
Ryr2 qF	ACGGCGACCATCCACAAAG	qPCR
Ryr2 qR	AAAGTCTGTTGCCAAATCCTTCT	qPCR
Cacna1c qF	CCTGCTGGTGGTTAGCGTG	qPCR
Cacna1c qR	TCTGCCTCCGTCTGTTTAGAA	qPCR
Cacna2d1 qF	GTCACACTGGATTTTCTCGATGC	qPCR

Cacna2d1 qR	GGGTTTCTGAATATCTGGCCTGA	qPCR
mtDNA1 qF	CTCTTATCCACGC TTCCGTTACG	qPCR
mtDNA1 qR	GATGGTGGTACTCCCGCTGTA	qPCR
mtDNA2 qF	CCCATTCCACTTCTGATTACC	qPCR
mtDNA2 qR	ATGATAGTA GAGTTGAGTAGCG	qPCR
H19 qF	GTACCCACCTGTCGTCC	qPCR
H19 qR	GTCCACGAGACCAATGACTG	qPCR
Mx1 qF	GACATAAGGTTAGCAGCTAAAGGATCA	qPCR
Mx1 qR	TCTCCGA TTAACCAGGCTAGCTAT	qPCR
Tnni3 Exon1 F	GGGGACCAGTGGGCAGGGGAACC	ChIP-qPCR
Tnni3 Exon1 R	GGCGTGTGGCTCGGTGGCATAGG	ChIP-qPCR
Tnni3 Exon6 F	CAATCTCCAGCCTCCCTCTCAGCATCCAC	ChIP-qPCR
Tnni3 Exon6 R	TCCAGGCTTCCCCGGGCTCCC	ChIP-qPCR
Mylk3 -4.5Kb F	GCAAACCTGGTCAAACTCAAAGCAAATG TATG	ChIP-qPCR
Mylk3 -4.5Kb F	TGTGTGCAGGCTGGATAGTAGAGGAGTT GG	ChIP-qPCR
Mylk3 Exon13 F	AGGTCTCATATGCTGCTTGCTCCCCCG	ChIP-qPCR
Mylk3 Exon13 R	CCTAATTCCTCCCTCTTCCATACCTCACTC CCTAC	ChIP-qPCR
Myocd -532bp F	AGAACGCGCTGGAGCCTGGGCC	ChIP-qPCR
Myocd -532bp R	TGAATCCAAAGAGAACATCCAACCACAG GG	ChIP-qPCR
Myocd Intron1 F	GTGCATGGCTGACCCCGGTGGC	ChIP-qPCR
Myocd Intron1 R	TGGATGGGACACTCTGGGGATTTCTTGAA	ChIP-qPCR
Myocd Exon14 F	GACAGCCCCATCGGAAAGGTGAGCG	ChIP-qPCR
Myocd Exon14 R	GGGAGGTGACAACTGTGCATGCATCGG	ChIP-qPCR
Dus1l Exon1 F	CCCAGAGGTGTTTGTCCAGGCGGCT	ChIP-qPCR
Dus1l Exon1 R	GGACCTCTCTTGGCTATCATCTGTGGGCA	ChIP-qPCR
Dus1l Exon10 F	CAGGCGCTAAAGCTTCGGTGTCAGGC	ChIP-qPCR
Dus1l Exon10 R	GTTGTCAGCTGGCCTCACTCCTTCTTGC	ChIP-qPCR
Snx19 TSS F	CTCCGATCCACCAGGCCGCTCTCC	ChIP-qPCR
Snx19 TSS R	GGAACGAACTCTTGGGGGGGTGGG	ChIP-qPCR
Snx19 Exon10 F	GGGAGTGAACAAGTGTCGGCTGAGCTGG	ChIP-qPCR
Snx19 Exon10 R	GCTCTGTCCCTACCAACCTTGCCACCC	ChIP-qPCR
Lsm6 TSS F	CGTTCTGACTGTTTTTGATCCCCGCGG	ChIP-qPCR

Lsm6 TSS R	GATCCAGAACGCTCACAGGCCGGG	ChIP-qPCR
Lsm6 Exon5 F	CTGCAGTTTTTCCCTCACGACCTTCAAGC	ChIP-qPCR
Lsm6 Exon5 R	GGGAAACACTCATAACTGCTTGGCCAAC TACA	ChIP-qPCR
Cpt1b flox F	GTGATGGCAATTATAGGCTAGTG	Genotyping
Cpt1b flox R	CTCCAGCCCCCAATCTCTAT	Genotyping
Cre F	GACCAGGTTCGTTCACTCATGG	Genotyping
Cre R	AGGCTAAGTGCCTTCTCTACAC	Genotyping

Supplementary Table 2: List of antibodies used in this study

Antibody	Use	Use Supplier	Cat. No.
α-Actinin (Sarcomere)	IF (1:500)	Sigma-Aldrich	A7811
Ki67	IF (1:500)	Abcam	ab15580
cTnT-FITC	IF (1:1000)	Abcam	ab105439
pH3	IF (1:500)	Millipore	06-570
Cyclin E1	WB (1:1000)	Abcam	ab7959
Aurora B	IF (1:200)	Abcam	Ab2254
CPT1B	WB (1:2000)	Proteintech	22170-1-AP
pan-actin	WB (1:5000)	Cell Signaling	4968
WGA, Alexa FluorTM 488	IF (1:500)	ThermoFisher Scienticfic	W11261
PCM1	FACS (1:500)	Sigma-Aldrich	HPA023374-100UL
PDH1a	WB (1:2000)	Proteintech	18068-1-AP
PDK4	WB (1:1000)	Proteintech	12949-1-AP
ACSS1	WB (1:1000)	Proteintech	17138-1-AP
ACSS2	WB (1:1000)	GeneTex	GTX30020
Acly	WB (1:1000)	Proteintech	18068-1-AP
IDH1	WB (1:2000)	Biorbyt	orb37973
IDH2	WB (1:2000)	ThermoFisher Scienticfic	MA5-17271
IDH3a	WB (1:2000)	Abcam	ab58641
OGDH	WB (1:2000)	Sigma-Aldrich	HPA020347-100UL
DLST	WB (1:1000)	Cell Signaling	5556
CS	WB (1:1000)	Proteintech	16131-1-AP
MDH2	WB (1:1000)	Proteintech	15462-1-AP
SDHC	WB (1:1000)	Proteintech	14575-1-AP
FH	WB (1:1000)	Proteintech	11375-1-AP
SDHB	WB (1:1000)	Proteintech	10620-1-AP
SDHA	WB (1:1000)	Cell Signaling	5839
ACO2	WB (1:1000)	Cell Signaling	6922
DLD	WB (1:1000)	ThermoFisher Scienticfic	PA5-27367
Phospho-Histone H2A.X (Ser139)	IF (1:500)	Cell Signaling	2577
Dihydrorhodamine 123	IF (1:200)	Invitrogen	11510346

Detyrosinated alpha Tubulin	IF (1:500)	Abcam	ab48389
H3K4me1	WB (1:1000)	Abcam	ab8895
H3K4me2	WB (1:1000)	Active Motif	39141
H3K4me3	WB (1:1000)	Millipore	07-473
H3K9me1	WB (1:1000)	Abcam	ab8896
H3K9me2	WB (1:1000)	Abcam	ab1220
H3K9me3	WB (1:1000)	Abcam	ab8898
H3K27me3	WB (1:1000)	Millipore	07-449
H3K36me3	WB (1:1000)	Abcam	ab9050
H3K79me2	WB (1:1000)	Millipore	04-835
H3K79me3	WB (1:1000)	Abcam	ab2621
H4K20me1	WB (1:1000)	Abcam	ab9051
H4K20me2	WB (1:1000)	Abcam	ab9052
H4K20me3	WB (1:1000)	Abcam	ab9053
H3	WB (1:5000)	Abcam	ab1791
H3K4me3	ChIP-seq (1:100)	Diagenode	C15410003-50
KDM5B	ChIP (1:50)	Cell Signaling	15327S
Goat anti-Mouse IgG (H+L) Alexa Fluor 488	IF, FACS (1:1000)	ThermoFisher Scienticfic	A28175
Goat anti-Rabbit IgG (H+L) Alexa Fluor 594	IF, FACS (1:1000)	ThermoFisher Scienticfic	A-11037

SUPPLEMENTARY FIGURES

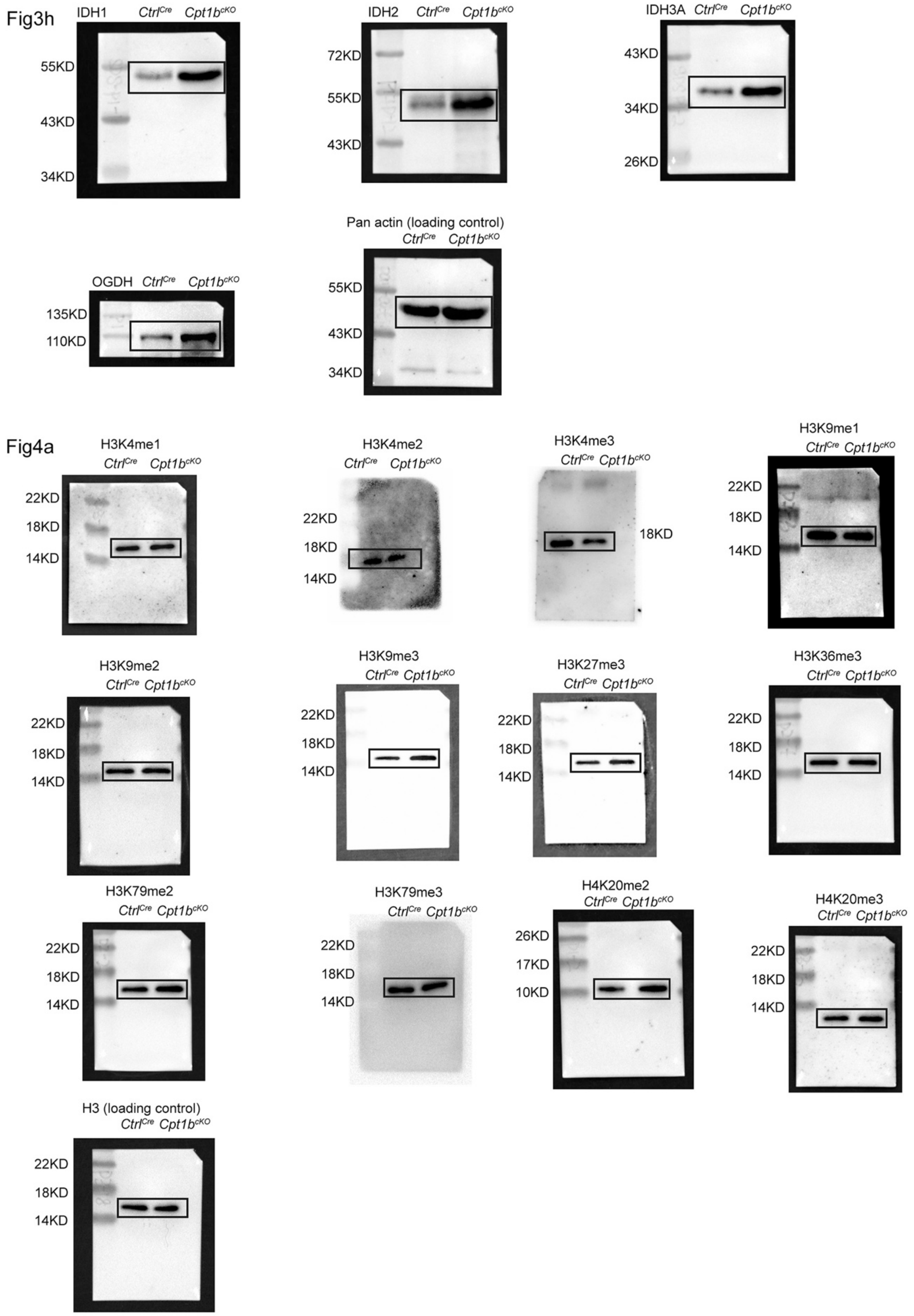

Supplementary Figure 1: Images of unprocessed blots used for Fig. 3h and Fig. 4e.

Fig5d

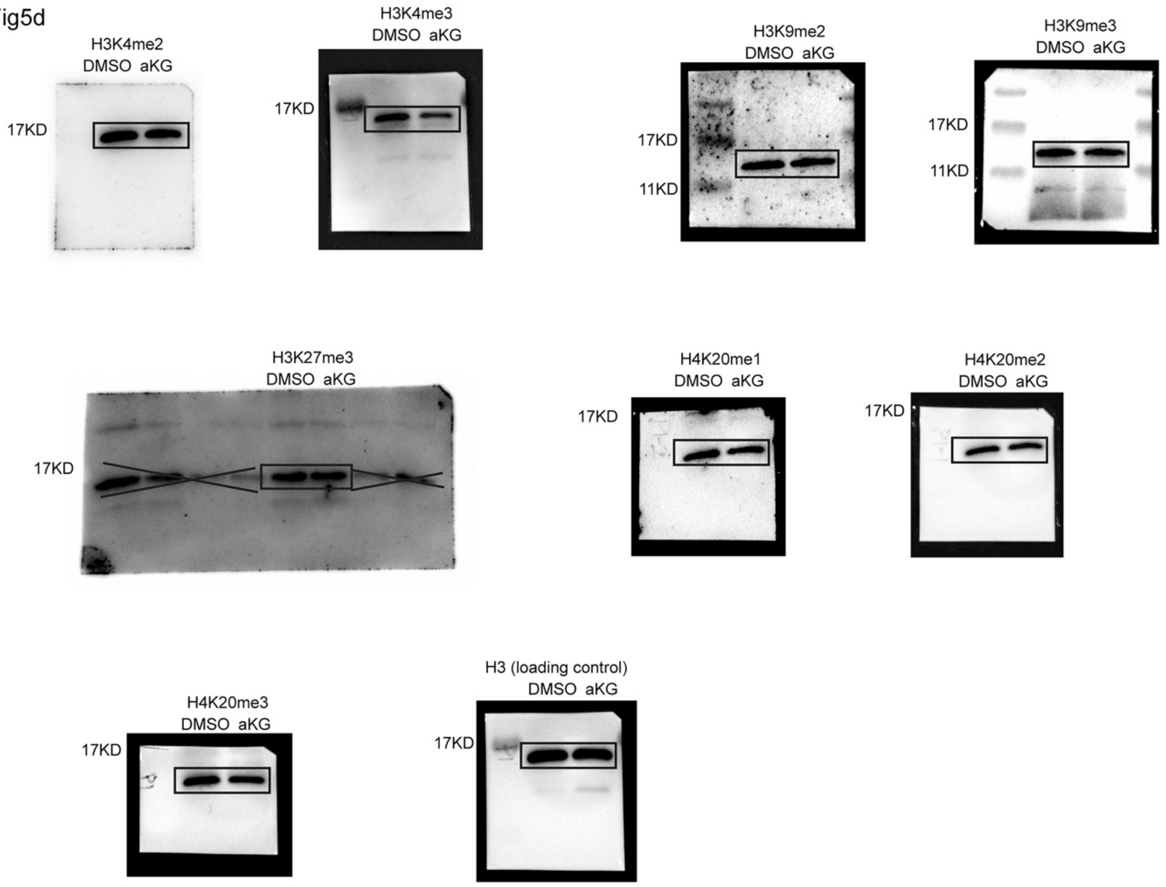

Fig5e

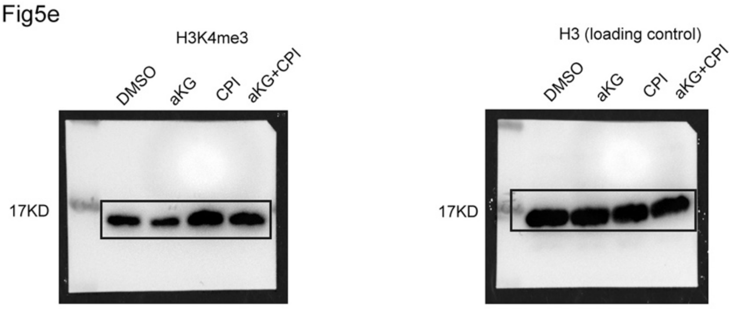

Supplementary Figure 2: Images of unprocessed blots used for Fig. 5d and Fig. 5e.

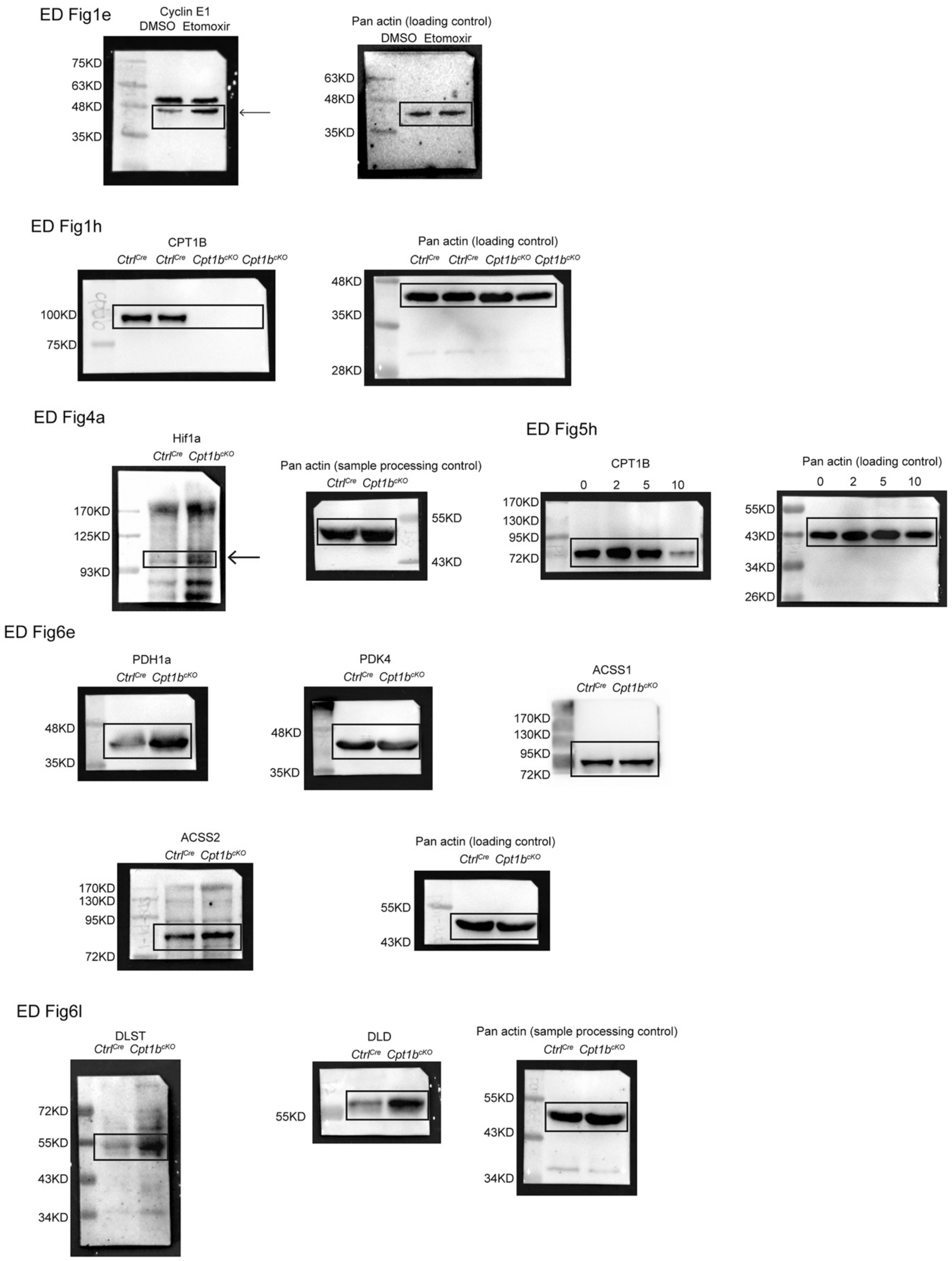

Supplementary Figure 3: Images of unprocessed blots used for Extended Figs.1e, 4a, 5h, 6e and 6l.

ED Fig6m

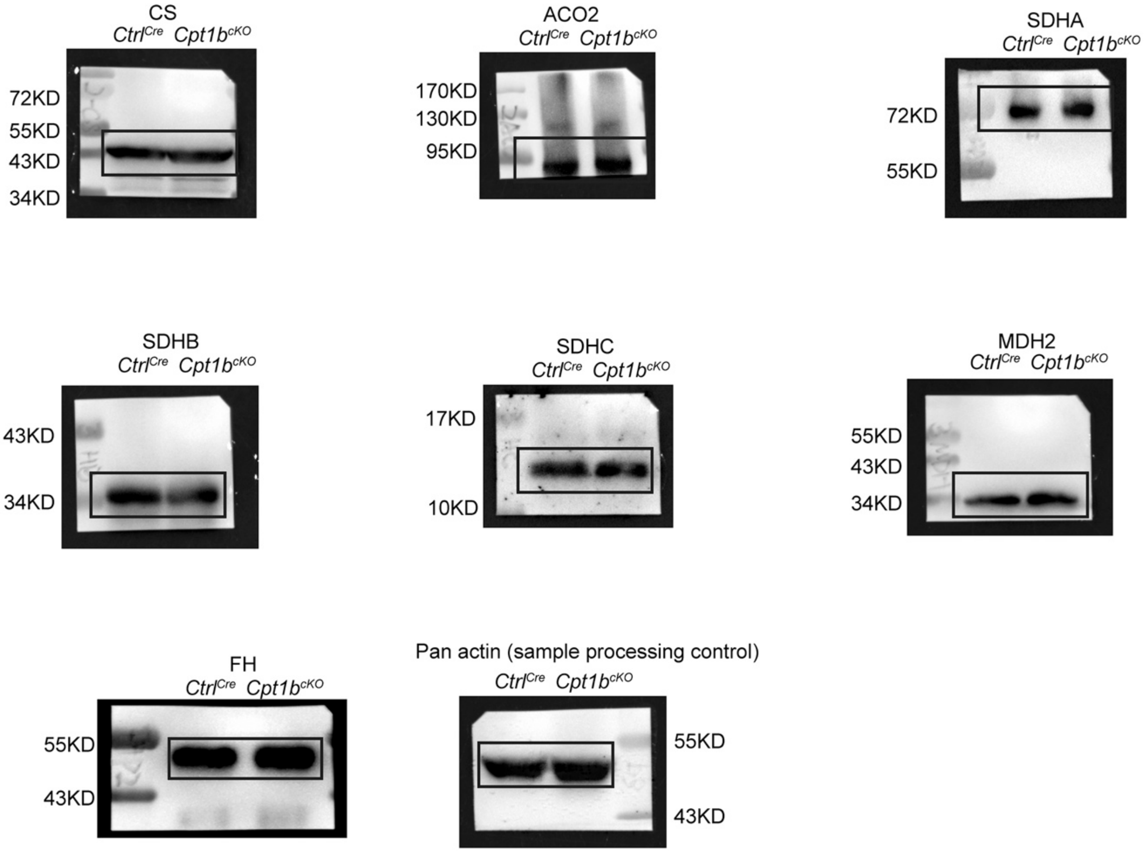

ED Fig7g

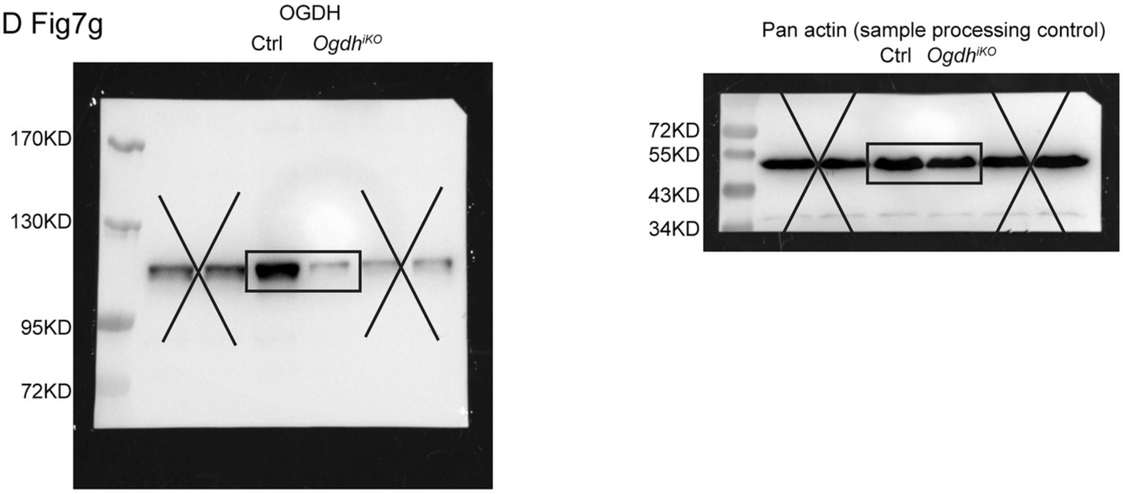

ED Fig8b

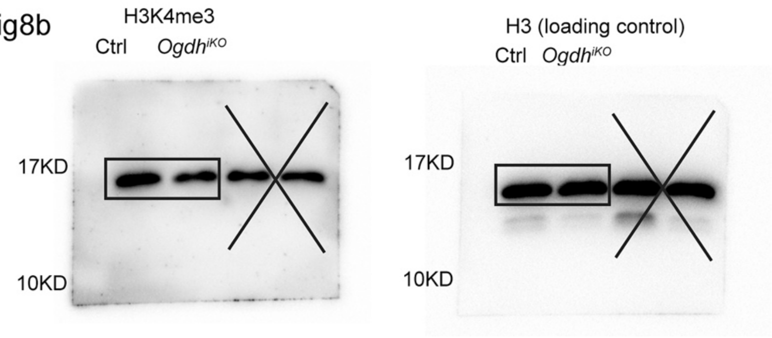

Supplementary Figure 4: Images of unprocessed blots used for Extended Figs.6m, 7g, and 8b.

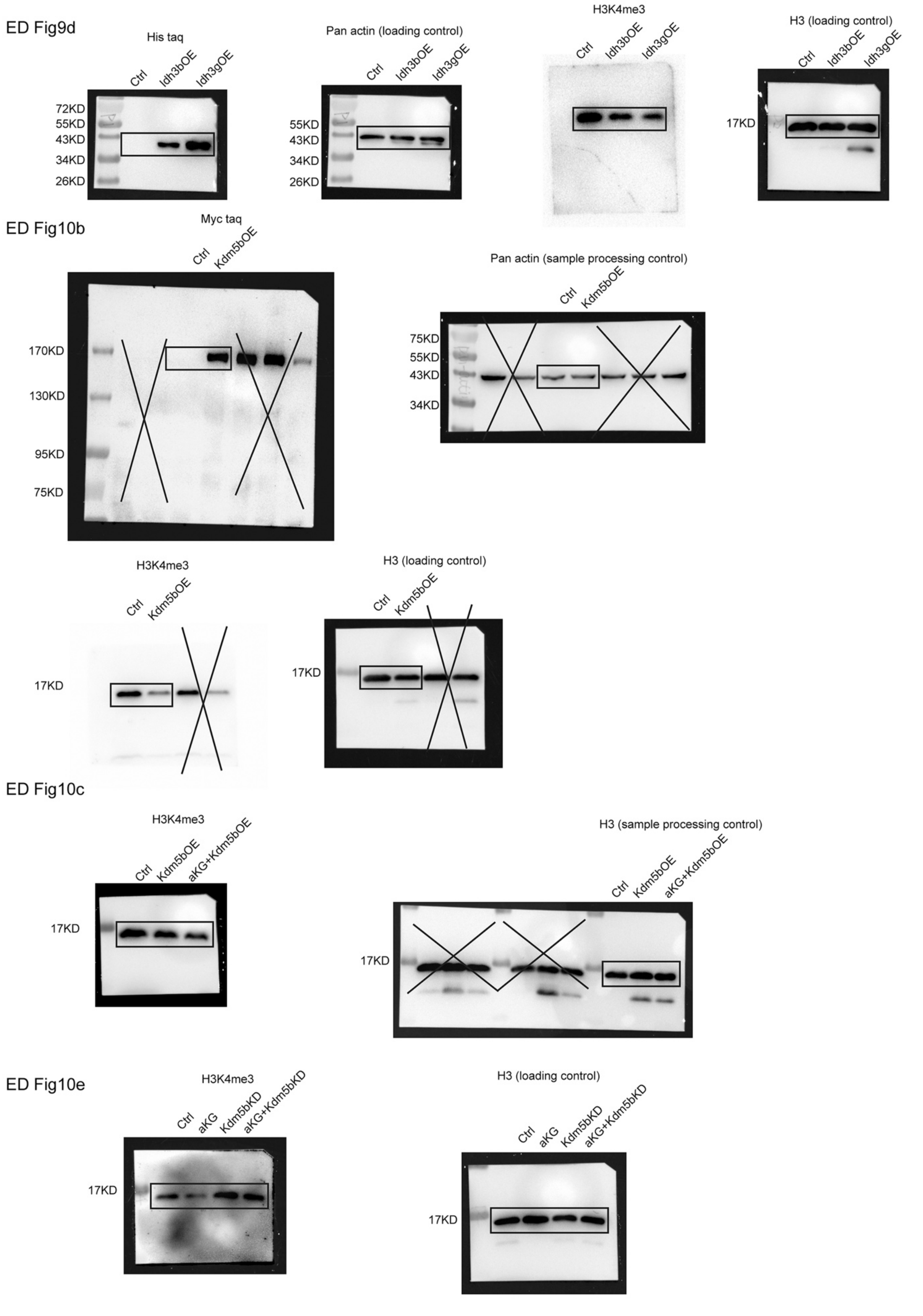

Supplementary Figure 5: Images of unprocessed blots used for Extended Figs. 9d, 10b, 10c, and 10e.

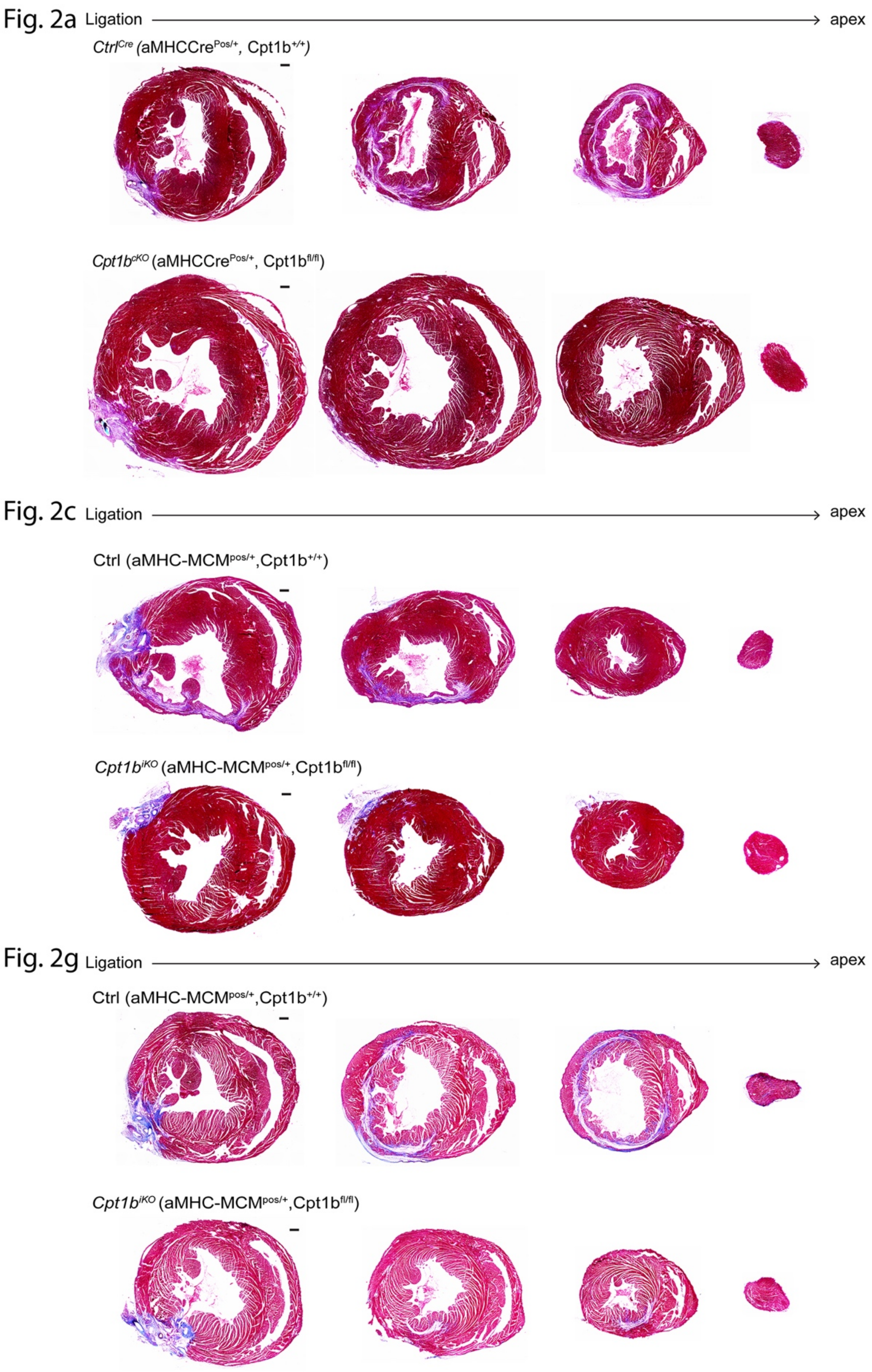

Supplementary Figure 6 (Page 16): Unprocessed images of trichrome-stained tissue sections from control and *Cpt1b*-deficient hearts, 3 weeks after myocardial infarction, used for Figs. 2a, 2c, and 2g.

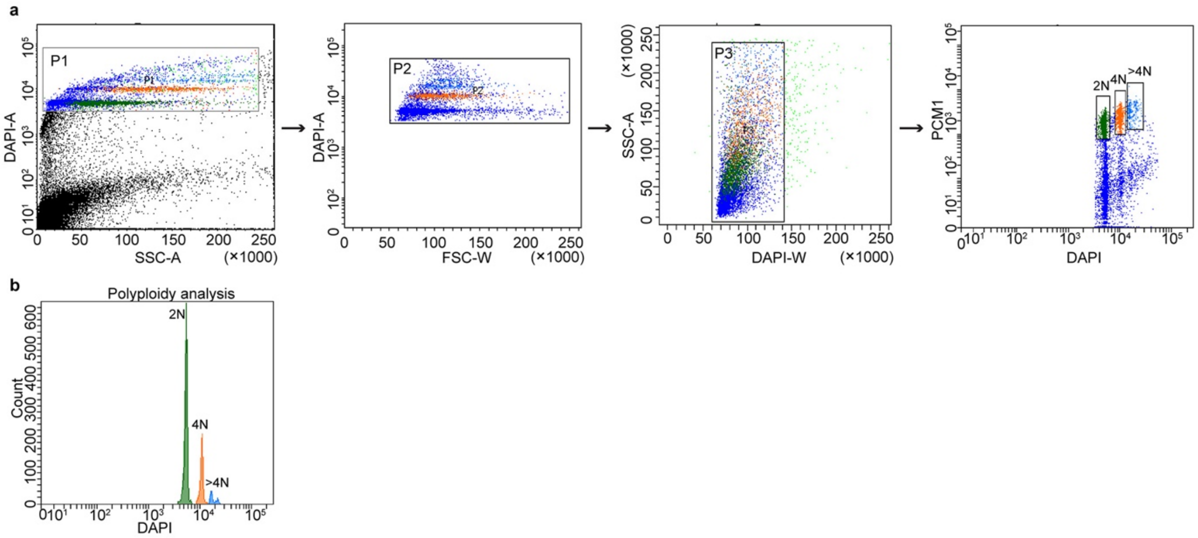

Supplementary Figure 7: Depiction of the FACS-gating strategy used for isolation of PCM1-positive cardiomyocyte nuclei.
