# Supplementary material for: Inhibition of fatty acid oxidation enables heart regeneration in adult mice
Source: Nature. 2023 Sep 27;622(7983):619–26. doi: 10.1038/s41586-023-06585-5 (PMC10584682; doi:10.1038/s41586-023-06585-5)
Supplement: Supplementary file 2 — Reporting Summary [file 41586_2023_6585_MOESM2_ESM.pdf]

nature portfolio

Corresponding author(s): Xuejun Yuan and Thomas Braun

Last updated by author(s): Jul 21, 2023

Reporting Summary

Nature Portfolio wishes to improve the reproducibility of the work that we publish. This form provides structure for consistency and transparency in reporting. For further information on Nature Portfolio policies, see our Editorial Policies and the Editorial Policy Checklist.

Statistics

For all statistical analyses, confirm that the following items are present in the figure legend, table legend, main text, or Methods section.

n/a	Confirmed	
☐	☒	The exact sample size (*n*) for each experimental group/condition, given as a discrete number and unit of measurement
☐	☒	A statement on whether measurements were taken from distinct samples or whether the same sample was measured repeatedly
☐	☒	The statistical test(s) used AND whether they are one- or two-sided<br>*Only common tests should be described solely by name; describe more complex techniques in the Methods section.*
☒	☐	A description of all covariates tested
☐	☒	A description of any assumptions or corrections, such as tests of normality and adjustment for multiple comparisons
☐	☒	A full description of the statistical parameters including central tendency (e.g. means) or other basic estimates (e.g. regression coefficient) AND variation (e.g. standard deviation) or associated estimates of uncertainty (e.g. confidence intervals)
☐	☒	For null hypothesis testing, the test statistic (e.g. *F*, *t*, *r*) with confidence intervals, effect sizes, degrees of freedom and *P* value noted<br>*Give P values as exact values whenever suitable.*
☒	☐	For Bayesian analysis, information on the choice of priors and Markov chain Monte Carlo settings
☒	☐	For hierarchical and complex designs, identification of the appropriate level for tests and full reporting of outcomes
☒	☐	Estimates of effect sizes (e.g. Cohen's *d*, Pearson's *r*), indicating how they were calculated

Our web collection on statistics for biologists contains articles on many of the points above.

Software and code

Policy information about availability of computer code

Data collection	ZEN 2 Imaging Software (ZEISS Software), ChemiDoc Imaging Systems (Biorad), Medis Imaging Systems, Leica SP8 - software LAS X 3.5.7.23225, FACS Aria TM III (BD Biosciences) - BD FACS Diva v8 Software, All the sequencing data were collected using Illumina Nextseq500 platform, Agilent Seahorse XFe96 Analyzer
Data analysis	Prism 9 software package, FastQC 0.11.8 (http://www.bioinformatics.babraham.ac.uk/projects/fastqc), Trimmomatic version >=0.36 (http://www.usadellab.org/cms/?page=trimmomatic), STAR >=2.5.4b, DESeq2 version >=1.14.0, Picard 2.21.7 (https://github.com/broadinstitute/picard/releases/tag/2.21.7), MUSIC peakcaller (version of Dec. 2015), bigWigAverageOverBed (UCSC Toolkit), IGV 2.3.52<br>Codes used in the study been deposited at github and are available under 'https://github.com/loosolab/Li_et_al_2023_heart_regeneration' (DOI: 10.5281/zenodo.7828994)

For manuscripts utilizing custom algorithms or software that are central to the research but not yet described in published literature, software must be made available to editors and reviewers. We strongly encourage code deposition in a community repository (e.g. GitHub). See the Nature Portfolio guidelines for submitting code & software for further information.

Data

Policy information about availability of data

All manuscripts must include a data availability statement. This statement should provide the following information, where applicable:

- Accession codes, unique identifiers, or web links for publicly available datasets
- A description of any restrictions on data availability
- For clinical datasets or third party data, please ensure that the statement adheres to our policy

Data have been deposited in public data bases. The sequencing reads were aligned versus mouse genome version mm10 (GRCm38.p5). Sequencing data are available at https://www.ncbi.nlm.nih.gov/geo/ under the accession number GSE172415 (https://www.ncbi.nlm.nih.gov/geo/query/acc.cgi?acc=GSE172415) and include results from ChIP-seq and RNA-seq experiments. RNA-seq data of neonatal CMs treated with DMSO and aKG have the accession number: GSE217188 (https://www.ncbi.nlm.nih.gov/geo/query/acc.cgi?acc=GSE217188).

Research involving human participants, their data, or biological material

Policy information about studies with human participants or human data. See also policy information about sex, gender (identity/presentation), and sexual orientation and race, ethnicity and racism.

Reporting on sex and gender	Not applicable
Reporting on race, ethnicity, or other socially relevant groupings	Not applicable
Population characteristics	Not applicable
Recruitment	Not applicable
Ethics oversight	Not applicable

Note that full information on the approval of the study protocol must also be provided in the manuscript.

Field-specific reporting

Please select the one below that is the best fit for your research. If you are not sure, read the appropriate sections before making your selection.

☒ Life sciences ☐ Behavioural & social sciences ☐ Ecological, evolutionary & environmental sciences

For a reference copy of the document with all sections, see nature.com/documents/nr-reporting-summary-flat.pdf

Life sciences study design

All studies must disclose on these points even when the disclosure is negative.

Sample size	Sample size were determined based on established practice and applicable standards. We opted for sample sizes which are commonly used sample size in the field. For in vivo studies, a minimum of three biological replicates were analyzed. For In vitro studies, experiments in which data were not quantified were performed with at least two replicates. Each experiment in which data were quantified was performed with at least 3 replicates.
Data exclusions	For both the in vivo and in vitro experiments, all the attempts were successfully and all the data were included during data processing.
Replication	All in vivo studies were performed with indicated number of animals. The in vitro study were performed at least 3 times independently and each replicate was successful. Sample sizes, statistical analyses and significance levels are all indicated in the figure legends or the method part.
Randomization	Animals were assigned to different groups according to genotypes. The genotype of animals from which individual samples was not known and experiments were performed in a blinded pattern. After data collection, individual genotypes were revealed and the animals were assigned to separate groups for further statistical analysis. For in vitro experiments, the samples were allocated into groups randomly.
Blinding	For in vivo study, all animals were numbered and experiments were performed in a blinded pattern. After data collection, genotypes were revealed and animals assigned to groups based on genotype for data analysis. In vitro experiments were not blinded during data collection or analysis since we know the treatment of each group before data collection. Positive controls, negative controls and samples were analyzed in exactly the same manner.

Reporting for specific materials, systems and methods

We require information from authors about some types of materials, experimental systems and methods used in many studies. Here, indicate whether each material, system or method listed is relevant to your study. If you are not sure if a list item applies to your research, read the appropriate section before selecting a response.

Materials & experimental systems

n/a	Involved in the study
☐	☒ Antibodies
☐	☒ Eukaryotic cell lines
☒	☐ Palaeontology and archaeology
☐	☒ Animals and other organisms
☒	☐ Clinical data
☒	☐ Dual use research of concern
☒	☐ Plants

Methods

n/a	Involved in the study
☐	☒ ChIP-seq
☐	☒ Flow cytometry
☒	☐ MRI-based neuroimaging

Antibodies

Antibodies used

α-Actinin (Sarcomere) 1:500 (Sigma-Aldrich A7811); Ki67 1:500 (Abcam ab15580); cTnT-FITC 1:1000 (Abcam ab105439); pH3 1:500 (Millipore 06-570); Ccne1 1:1000 (Abcam ab7959); Aurora B 1:200(Abcam Ab2254); Cpt1b 1:2000(Proteintech 22170-1-AP); pan-actin 1:5000(Cell Signaling 4968); WGA Alexa FluorTM 488 1:500 (ThermoFisher Scienticfic W11261); PCM1 1:500 (Sigma-Aldrich HPA023374-100UL); PDH1a 1:2000(Proteintech 18068-1-AP); PDK4 1:1000(Proteintech 12949-1-AP); ACSS1 1:1000(Proteintech 17138-1-AP); ACSS2 1:1000 (GeneTex GTX30020); ACL 1:1000 (Proteintech,18068-1-AP); IDH1 1:2000 (Biorbyt orb135710); IDH2 1:2000 (ThermoFisher Scienticfic MA5-17271); IDH3a 1:2000(Abcam ab58641); OGDH 1:2000(Sigma-Aldrich HPA020347-100UL); DLST 1:1000(Cell Signaling 5556); DLD 1:1000(ThermoFisher Scienticfic PA5-27367); CS 1:1000(Proteintech, 16131-1-AP); MDH2 1:1000 (Proteintech, 15462-1-AP); SDHC 1:1000 (Proteintech, 14575-1-AP); FH 1:1000 (Proteintech,11375-1-AP); SDHB 1:1000 (Proteintech, 10620-1-AP); SDHA 1:1000 (Cell Signaling, 6922); ACO2 1:1000 (Cell Signaling, 5839); Phospho-Histone H2A.X (Ser139) 1:500 (Cell Signaling, 2577); Detyrosinated alpha Tubulin 1:500 (Abcam, ab48389); H3K4me1 1:1000 (Abcam ab8895); H3K4me2 1:1000 (Active Motif 39141); H3K4me3 1:1000 (Millipore 07-473); H3K9me1 1:1000 (Abcam ab8896); H3K9me2 1:1000 (Abcam ab12200); H3K9me3 1:1000 (Abcam ab8898); H3K27me3 1:1000 (Millipore 07-449); H3K36me3 1:1000 (Abcam ab9050); H3K79me2 1:1000 (Millipore 04-835); H3K79me3 1:1000 (Abcam ab2621); H4K20me1 1:1000 (Abcam ab9051); H4K20me2 1:1000 (Abcam ab9052); H4K20me3 1:1000 (Abcam ab9053); H3 1:5000 (Abcam ab1791); H3K4me3 1:100 (Diagenode C15410003-50); KDM5B 1:50 (Cell Signaling 15327S); Goat anti-Mouse IgG (H+L) Alexa Fluor 488 1:1000 (ThermoFisher Scienticfic A28175); Goat anti-Rabbit IgG (H+L) Alexa Fluor 594 1:1000 (ThermoFisher Scienticfic A-11037).

Validation

α-Actinin (Sarcomere) has been validated with mouse sample by Luo et al, Circ Res, 2017. Ki67, pH3, AuroraB, PCM1 was validated by Chen et al, Science , 2021. Ccne1 has been validated with mouse sample in WB by Cang Y, et al, Cell, 2006. Cpt1b was validated with mouse sample in WB by Simcox, et al, Cell Metab, 2017. Pan-actin was validated with mouse sample in WB by Hillege, et al, Elife, 2022. WGA was validated in IF with mouse heart tissue by Chatterjee et al, Cell, 2016. OGDH, DLD, DLST were validated with mouse sample in WB by Andrade J, et al, Nat Cell Biol, 2021. PDH1a, PDK4, IDH1 were validated with mouse kidney tissue lysate via information on manufacturer's website. ACSS1 was validated in WB with mouse sample by Odera JO, et al, Biochem J, 2020. ACSS2 was validated with mouse liver lysate with information from manufacturer's website. IDH2, IDH3a was validated with mouse brain extracts with information from manufacturer's website. CS was validated with mouse sample in WB by Xu Yj, et al, EMBO J, 2021. MDH2 antibody was validated with mouse sample by Sinha T, et al, iScience, 2020. SDHC antibody was validated with mouse sample in WB by Kauppila JH, et al, Nucleic Acids Res, 2018. FH antibody was validated with mouse sample in WB by Yuan MH, et al, Aging Dis, 2022. SDHB antibody was validated with mouse sample in Ding M, et al, Diabetes, 2022. ACO2 antibody was validated with mouse sample via Silvia V, et al, Cell Rep, 2021. Phospho-Histone H2A.X (Ser139) was validated in IF by Ratnaparkhe M, et al, Nat Commun, 2018. Detyrosinated alpha Tubulin was validated with mouse heart sample by Schuldt M, et al, Front Cardiovasc Med, 2021. All histone antibody, includes H3K4me1, H3K4me2, H3K4me3, H3K27me3, H3K79me2 and H4K20me1, H3K9me1, H3K9me2, H3K9me3, H3K36me3 and H3K79me3, H4K20me2 and H4k20me3, H3 was validated Interactive Database for the Assessment of Histone Antibody Specificity. KDM5B was validated with extracts from K-562cell lines in ChIP experiment.

Eukaryotic cell lines

Policy information about cell lines and Sex and Gender in Research

Cell line source(s)	HEK293T from ATCC
Authentication	Authenticated human cell lines were obtained from ATCC and maintained as instructed. The cell was checked for morphology for cell authentication. .
Mycoplasma contamination	mycoplasma free
Commonly misidentified lines (See ICLAC register)	No commonly misidentified cell lines were used

Animals and other research organisms

Policy information about studies involving animals; ARRIVE guidelines recommended for reporting animal research, and Sex and Gender in Research

Laboratory animals	All mice were maintained on a C57BL/6 background, and littermates were used as controls in all experiments. All mice were maintained in individually ventilated cages, at 22.5 °C ± 1 °C and a relative humidity of 50% ± 5% with controlled illumination (12 h dark/light cycle). Mice were given ad libitum access to food and water. All experiments were performed on balanced cohorts of male and female mice. In experiments, in which genes were inactivated by Cre recombinase-mediated recombination, corresponding Cre recombinase-expressing strains without the floxed target genes were always used as negative controls. αMHC-MCM control mice were subjected to the same tamoxifen treatment as in the actual gene inactivation experiment
Wild animals	Studies did not involve wild animals.
Reporting on sex	Observed results did not differ between male and female mice. No gender-specific experiments were performed.
Field-collected samples	Studies did not involve samples collected in the field.
Ethics oversight	All animal experiments were done in accordance with the Guide for the Care and Use of Laboratory Animals published by the US National Institutes of Health (NIH Publication No. 85-23, revised 1996) and were approved by the responsible Committee for Animal Rights Protection of the State of Hessen (Regierungspraesidium Darmstadt, Wilhelminenstr. 1-3, 64283 Darmstadt, Germany) with the project number B2/1125, B2/1137, B2/1056 and B2/2034.

Note that full information on the approval of the study protocol must also be provided in the manuscript.

Plants

Seed stocks	Not applicable
Novel plant genotypes	Not applicable
Authentication	Not applicable

ChIP-seq

Data deposition

☒ Confirm that both raw and final processed data have been deposited in a public database such as GEO.

☒ Confirm that you have deposited or provided access to graph files (e.g. BED files) for the called peaks.

Data access links<br>*May remain private before publication.*	https://www.ncbi.nlm.nih.gov/geo/query/acc.cgi?acc=GSE172415
Files in database submission	GSM5255555 Input-CtrlCre_1<br>GSM5255556 Input-CtrlCre_2<br>GSM5255557 Input-CtrlCre_3<br>GSM5255558 Input-Cpt1bcKO_1<br>GSM5255559 Input-Cpt1bcKO_2<br>GSM5255560 Input-Cpt1bcKO_3<br>GSM5255561 ChIP-CtrlCre_1<br>GSM5255562 ChIP-CtrlCre_2<br>GSM5255563 ChIP-CtrlCre_3<br>GSM5255564 ChIP-Cpt1bcKO_1<br>GSM5255565 ChIP-Cpt1bcKO_2<br>GSM5255566 ChIP-Cpt1bcKO_3
Genome browser session<br>(e.g. UCSC)	N/A

Methodology

Replicates	Three biological replicates for H3K4me3 ChIP-seq.
Sequencing depth	Input-CtrlCre_1 27M 80% aligned<br>Input-CtrlCre_2 20M 79% aligned<br>Input-CtrlCre_3 32M 79% aligned<br>Input-Cpt1bcKO_1 29M 79% aligned<br>Input-Cpt1bcKO_2 35M 80% aligned<br>Input-Cpt1bcKO_3 25M 78% aligned<br>ChIP-CtrlCre_1 26M 79% aligned

ChIP-CtrlCre_2 25M 79% aligned
ChIP-CtrlCre_3 26M 78% aligned
ChIP-Cpt1bcKO_1 31M 80% aligned
ChIP-Cpt1bcKO_2 30M 81% aligned
ChIP-Cpt1bcKO_3 31M 79% aligned
Single end.

Antibodies

H3K4me3 (Diagenode C15410003-50)

Peak calling parameters

The MUSIC peakcaller (version of Dec. 2015) was employed in punctate mode to identify enriched regions when comparing the respective ChIP to input samples. The MUSIC FDR was set to 0.2. Peaks overlapping ENCODE blacklisted regions (known misassemblies, satellite repeats) were excluded. In order to compare peaks in different samples for assessment of reproducibility, the resulting lists of significant peaks were overlapped and unified to represent identical regions. Sample counts for union peaks were produced using bigWigAverageOverBed (UCSC Toolkit) and normalized with DESeq2 1.26.0 to compensate for differences in sequencing depth, library composition, and efficiency. Peaks were annotated with the promoter of the nearest gene in range (TSS +- 5000 nt) using reference data of GENCODE vM15. Peaks were classified to be significantly differentially expressed with P-Value < 0.05 as produced by DESeq2. Peaks were divided into 3 groups based on peak length (Broad: >75th %, Medium: 75th – 25th %, Narrow: <25th %).

Data quality

id #	peaks raw
ChIP-Cre_1	16043
ChIP-Cre_2	10913
ChIP-Cre_3	16138
ChIP-KO_1	13312
ChIP-KO_2	16303
ChIP-KO_3	15843

Software

FastQC 0.11.8 (http://www.bioinformatics.babraham.ac.uk/projects/fastqc), Trimmomatic version >=0.36 (http://www.usadellab.org/cms/?page=trimmomatic), STAR >=2.5.4b, DESeq2 version >=1.14.0, Picard 2.21.7 (https://github.com/broadinstitute/picard/releases/tag/2.21.7), MUSIC peakcaller (version of Dec. 2015), bigWigAverageOverBed (UCSC Toolkit), IGV 2.3.52

Flow Cytometry

Plots

Confirm that:

☒ The axis labels state the marker and fluorochrome used (e.g. CD4-FITC).

☒ The axis scales are clearly visible. Include numbers along axes only for bottom left plot of group (a 'group' is an analysis of identical markers).

☒ All plots are contour plots with outliers or pseudocolor plots.

☒ A numerical value for number of cells or percentage (with statistics) is provided.

Methodology

Sample preparation

Ventricle were washed with ice-cold PBS after dissection and snap frozen in liquid N2. For cardiac nuclei isolation, the frozen ventricle was thawed in 3ml lysis buffer (5 mM CaCl2, 3 mM MgAc, 2 mM EDTA, 0.5 mM EGTA, and 10 mM Tris-HCl, pH 8) in M tube (Miltenyi Biotec) and homogenized by the gentleMACS Dissociator (Miltenyi Biotec) following the manufacturer's protocol (protein_01). The resultant homogenate was mixed with lysis buffer containing 0.4% Triton X-100, incubated on ice for 10 min, and subsequently filtered through 40um cell strainer (BD Bioscience). The flow-through was subjected to centrifugation at 1000 g for 5min at 4°C to harvest nuclei. Nuclei were further purified by centrifugation at 1000g for 5 min at 4°C through a 1M sucrose cushion (3 mM MgAc, 10 mM Tris-HCl, pH8) and then stained with a PCM1 antibody in nuclei stain buffer (DPBS, 1% BSA, 0.2% Igepal CA-630, 1 mM EDTA). DNA was stained by DAPI before FACS sorting.

Instrument

FACS sorting was done using a FACSAriaTM III (BD Biosciences).

Software

BD FACS Diva v8 software

Cell population abundance

Sorted cells were reanalyzed to assess purity. A 85% purity was achieved. The sorted PCM1 positive cardiac nuclei were further checked under immunofluorescence microscope to confirm the purity.

Gating strategy

The detailed gating strategy was shown in Supplementary Figure 7. In brief, the samples were firstly cleaned utilizing the SSC-A and DAPI-A to remove the DAPI-negative cell debris, the selected population was labeled as P1. The P1 population was further cleaned with cutoffs from DAPI-A and FSC-W, and the positive population was labeled as P2. The P2 population was then selected based on SSC-A and DAPI-W and the positive population was labeled as P3. Within the P3 population, the final population was selected based on the PCM1 signal intensity, the positive population was labeled and shown in Supplementary Figure 7. The PCM1 positive nuclei were further characterized into sub-clusters based on DNA content reflected by DAPI signal intensity.

☒ Tick this box to confirm that a figure exemplifying the gating strategy is provided in the Supplementary Information.
